# A mixed methods study of the impact of WAGR spectrum disorder on individuals and their caregivers

**DOI:** 10.1186/s13023-026-04360-z

**Published:** 2026-04-24

**Authors:** Erica V. Sistare, Andrew M. George, Evan R. Hathaway, Kelly L. Trout, John Morris, Shari McCullen Krantz, Jennifer M. Kalish

**Affiliations:** 1 Master’s Program in Genetic Counseling, Boston University Chobanian & Avedisian School of Medicine, Boston, MA USA; 2 Division of Genetic and Genomic Medicine, Children’s Hospital of Philadelphia, Philadelphia, PA USA; 3 Center for Childhood Cancer Research, Children’s Hospital of Philadelphia, Philadelphia, PA USA; 4 Department of Genetics and Pediatrics, Perelman School of Medicine, University of Pennsylvania, Philadelphia, PA USA; 5 International WAGR Syndrome Association, Montgomery Village, MD USA

**Keywords:** WAGR syndrome, WAGR spectrum, Lived experiences, Caregiving experience

## Abstract

**Background:**

WAGR spectrum disorder (WSD) is a rare genetic condition classically characterized by Wilms tumor, Aniridia, Genitourinary abnormalities, and Range of developmental delay. Following our publication, the terminology has shifted from “syndrome” to “spectrum disorder” to better capture its phenotypic variability.^1^ While other multi-systemic features have been identified, there is a paucity of literature around how WSD impacts individuals and their families. To guide clinical management recommendations and development of outcome measures for WSD clinical studies, this study explored the lived experiences of children with WSD and their caregivers.

**Methods:**

Fourteen caregivers to 13 children with WSD participated in this two-part study, consisting of a survey that collected medical information for each child with WSD and semi-structured interviews that focused on how WSD impacts daily life. Survey data were analyzed using descriptive statistics. Interview data were analyzed using reflexive thematic analysis within a critical realism framework.

**Results:**

Aniridia was the most frequently reported health condition, although a broad range of co-occurring conditions exemplified clinical heterogeneity of WSD. Analysis of caregiver interviews generated five key themes: (1) uncertainty has a profound emotional impact on caregivers, (2) while the prospect of lifelong caregiving is stressful, caring for a child with WSD is rewarding, (3) partnerships between healthcare providers and families are critical, (4) three areas to prioritize for assessment in future WSD clinical studies include aniridia/vision, behavioral/psychiatric, and speech/communication conditions, and (5) the WSD community is a powerful support system.

**Conclusions:**

This study offers valuable insight into the multifaceted impacts of WSD on individuals and their caregivers. Our findings highlight key therapeutic targets and opportunities to improve clinical care and support for the WSD community.

**Supplementary Information:**

The online version contains supplementary material available at 10.1186/s13023-026-04360-z.

## Background

WAGR spectrum disorder (WSD) is a rare microdeletion syndrome characterized by a wide range of clinical features, including an associated tumor predisposition [1, 2]. The term “WAGR” is derived from the condition’s classic features: Wilms tumor, Aniridia, Genitourinary abnormalities, and Range of developmental delay. In WSD, the deleted chromosomal region involves 11p13 and minimally includes the *WT1* and *PAX6* genes, which have been associated with Wilms tumor and aniridia, respectively. In view of the estimated 50% risk of Wilms tumor for individuals with WSD, age-specific tumor surveillance guidelines have been established, including regular abdominal or renal ultrasounds [3].

Analysis of phenotypic data from WSD cohorts has expanded the scope of classic WSD features and revealed additional features across multiple organ systems [1, 2]. In recognition of the clinical spectrum of this condition, WAGR syndrome was recently renamed “WAGR spectrum disorder” [1]. Deletion of other genes within or near the 11p13 region contributes to the wide range of clinical features within the WSD population. For example, approximately 50% of individuals with WSD have a heterozygous deletion of the *BDNF* gene, which has been associated with early-onset obesity, hyperphagia, and pain insensitivity [4, 5].

Despite several studies on the clinical characteristics of WSD, there is limited research regarding how WSD impacts the daily lives of individuals living with the condition and their caregivers. Existing surveys that have indirectly assessed this topic may not fully capture the nuanced experiences, needs, and values of the WSD community. Descriptions of a community’s lived experiences are critical for identifying interventions, resources, and future research directions that best support the community. The Food and Drug Administration (FDA) has recognized the importance of incorporating patient and caregiver perspectives into medical product development [6]. By centering the voices of affected communities, the FDA can approve products that yield clinical endpoints that are of most value to community members.

Within rare disease communities, one formal framework that has been used to identify meaningful, condition-specific clinical endpoints is the disease concept model (DCM) [7–11]. The development of a DCM yields a rigorous description of a rare disease and its impacts on members of the rare disease community. DCMs have primarily been developed by interviewing caregivers of individuals with a rare genetic condition to explore symptom impacts. After quantifying health conditions and other concepts discussed in qualitative interviews, the most frequently referenced health conditions are proposed as potential clinical endpoints. Prior DCMs have focused on genetic disorders that predominantly affect neurodevelopment, such as *STXBP1*-related disorders, Angelman syndrome, and *CACNA1A*-related hemiplegic migraine. In the present study, we leveraged tools of previous DCMs with important differences. We conducted interviews with caregivers of children with WSD that focused on similar topics from previous DCMs. However, we employed a different methodology for analyzing interview transcripts to better capture the nuanced experiences of the WSD community. To improve quantification of WSD symptomatology, we added a survey component inquiring about health conditions of children with WSD.

This study improves on previous DCM methodologies to provide a comprehensive description of the lived experiences of the WSD community. The findings of our study highlight clinical endpoints and holistic clinical management recommendations that are of highest priority to WSD community members.

## Methods

To investigate health conditions associated with WSD and the impacts of WSD on individuals and their families, this mixed methods study involved the verbal administration of a survey to caregivers of children with WSD and semi-structured interviews with these caregivers. The research team included several individuals with expertise in WSD. Two of the authors provide clinical care to individuals with WSD. Three members of the International WAGR Syndrome Association (IWSA), who are parents of children with WSD, were consulted in the conception, piloting, and administration of this study. This study was approved as an exempt protocol by the Boston University Medical Campus Institutional Review Board (Protocol H-45182) and by the Children’s Hospital of Philadelphia Institutional Review Board (Protocol 24-022554).

### Participants

Eligible participants were 18 years of age or older, felt comfortable speaking English, and self-identified as a caregiver to an individual with WSD between two and 18 years of age. Participants were recruited via email. Recruitment emails were sent to (1) WSD families who were enrolled in the Genetic Patient Registry study at the Children’s Hospital of Philadelphia and had opted in to learn about additional research studies and (2) families that were identified as being eligible for this study by the IWSA.

### Instrumentation

This study involved both a survey and a semi-structured interview. The survey collected medical information for each child with WSD along with demographic information for the child and their caregiver (Supplemental File 1). The list of health conditions included in the survey was based on those reported in Duffy et al. (2021) and a further review of the literature on WSD. The semi-structured interview guide (Supplemental File 2), focused on how WSD impacts the lives of individuals with WSD and their families, was adapted from prior studies directed to DCM development [9, 11].

### Procedures

Between October 2024 and February 2025, the first author administered the survey and conducted an interview via one Microsoft Teams meeting per family. Study meetings lasted between 55 and 124 min and were recorded. The survey and a list of interview topics were circulated to participants ahead of time. Survey data were collected live during the first part of each meeting, and interviews were conducted during the second portion. After completion of 13 interviews with 14 caregivers, the research team determined sufficient informational power was reached considering the specificity of the patient population, study aims, and depth of interview data [12]. In addition to the first author, at least one other member of the research team observed each participant meeting. The additional team member(s) created a written transcript of each interview by copying live captions from Microsoft Teams. Using audio files from the interviews, the first author and another member of the research team manually reviewed and edited interview transcripts to ensure verbatim accuracy. All transcripts were de-identified by the first author. As compensation, each family received a $25 Amazon gift card.

### Data analysis

The first author analyzed survey data using descriptive statistics. To synthesize caregiver perspectives and experiences from interview transcripts, reflexive thematic analysis was employed, an approach that acknowledges the subjective nature of qualitative analysis [13, 14]. A critical realism framework underpinned the analytical approach in this study [15]. Assumptions within this framework include that a WSD reality exists but that caregivers have their own subjective perceptions of WSD and relationships with their children across various social and cultural settings.

The first author coded interview transcripts using NVivo software, which involved assigning labels to capture ideas from the data. Both deductive coding and inductive coding were utilized. A priori codes were adapted from codes categorized as individual and caregiver impacts from a prior DCM study [11]. During the coding process, the first author consulted the research team to discuss and refine codes. Initial themes were generated from coded data, reviewed with the research team, and refined to generate final themes. Quotes were revised for removal of filler words and for clarity.

## Results

Fourteen caregivers from 13 families completed the combined survey and semi-structured interview. Demographic information of caregivers is summarized in Table 1. Most caregivers were white, women, employed full-time, and college-educated. Half of participants held a master’s degree or higher. Caregiver age spanned from early 30s to early 60s, with an average age of 43 years. The majority of caregivers were biological parents of children with WSD, with three members of the cohort identifying as an adoptive parent or kinship legal guardian. While most participating families lived in the United States, some lived in Europe.

**Table 1:** Demographics of caregivers (*n* = 14)

Item	*n* (%)
Age (years)	
30–40	5 (35.7)
41–51	8 (57.1)
52–62	1 (7.1)
Racial or ethnic identity	
Asian	1 (7.1)
White	13 (92.9)
Gender identity	
Man	2 (14.3)
Woman	12 (85.7)
Relationship to child with WSD	
Adoptive parent	2 (14.3)
Biological parent	11 (78.6)
Kinship legal guardian	1 (7.1)
Highest level of education	
Some college	2 (14.3)
Vocational/professional certificate	1 (7.1)
Bachelor’s degree	4 (28.6)
Master’s degree	6 (42.9)
Doctoral or other professional degree	1 (7.1)
Employment status	
Full-time	8 (57.1)
Part-time	4 (28.6)
Not currently working outside the home	2 (14.3)
Total children living in household (including child with WSD)	
1	2 (14.3)
2	10 (71.4)
3	2 (14.3)

Demographic information of participants’ children who have WSD (*n* = 13) is summarized in Table 2. Almost 70% of participants’ children with WSD were white, and most were girls. Ages of children with WSD ranged from two to 17 years, with a mean age of nine years. Most children with WSD lived with at least one sibling. Two children attended a residential schooling program on weekdays.

**Table 2:** Demographics of children with WSD (*n* = 13)

Item	*n* (%)
Age (years)	
2–5	2 (15.4)
6–9	6 (46.2)
10–13	2 (15.4)
14–17	3 (23.1)
Gender identity	
Boy	5 (38.5)
Girl	8 (61.5)
Racial or ethnic identity	
Asian	2 (15.4)
More than one race or ethnicity selected	2 (15.4)
White	9 (69.2)

**Fig. 1:**
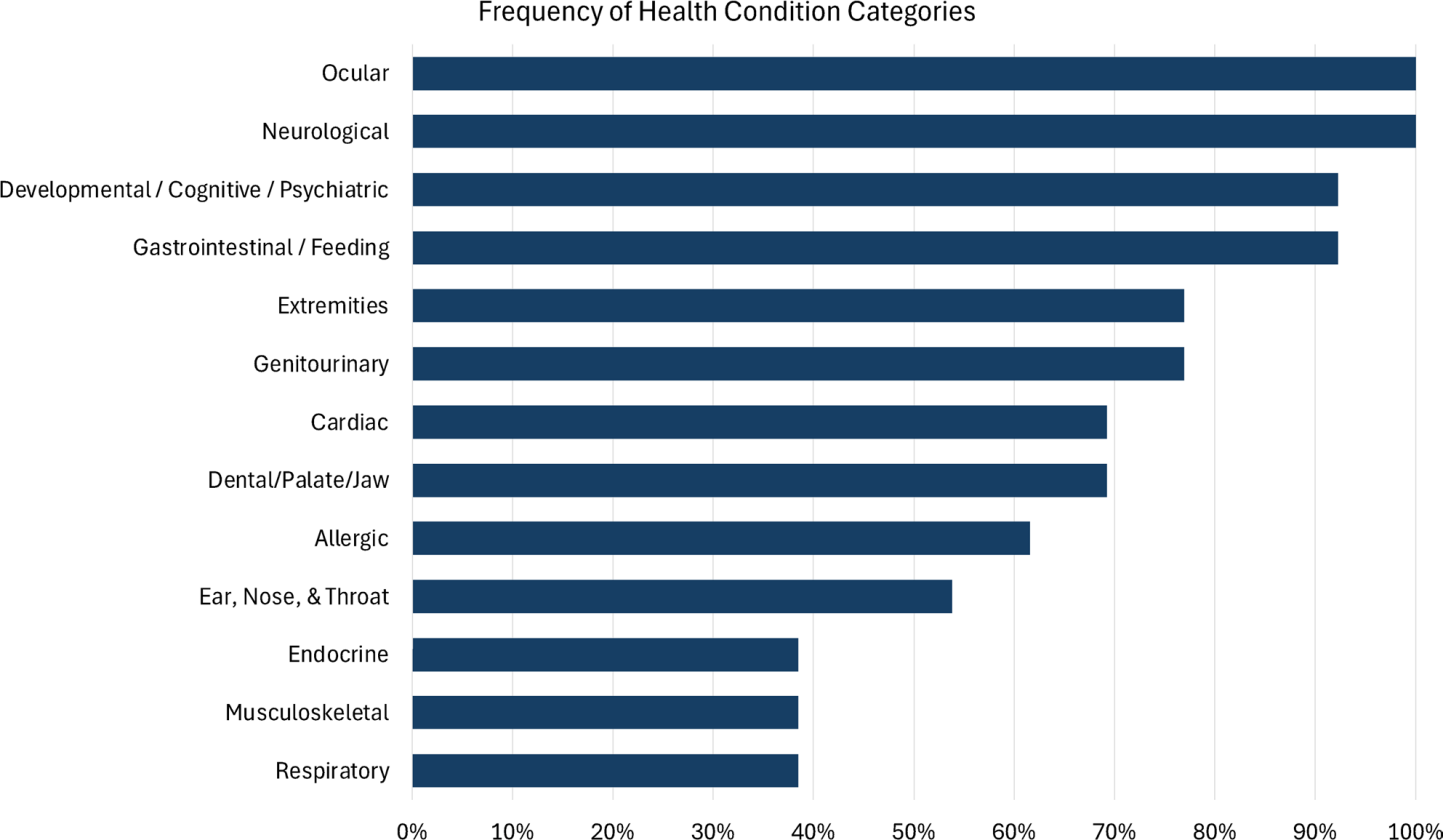
Frequency of health conditions for children with WSD by category

Among children with WSD in this cohort, health conditions spanned several organ systems. The frequencies of health condition categories are displayed in Fig. 1, with the frequency of specific health conditions within each category shown in Supplemental File 3. All children had at least one ocular condition and one neurological condition. Aniridia was the only shared health condition among all 13 children with WSD in this study. In addition to aniridia, all children had at least one other ocular finding, with nystagmus, cataracts, and glaucoma being the next most common ocular conditions. In terms of neurological conditions, pain insensitivity and muscle tone differences predominated. Approximately 75% of children with WSD had at least one condition in the Developmental/Cognitive/Psychiatric, Extremities, Gastrointestinal/Feeding, and Genitourinary categories. Approximately 54% of children with WSD had a history of Wilms tumor and/or nephrogenic rests. One child had a history of multiple primary Wilms tumors. No history of Wilms relapse was reported in this cohort. Developmental history ranged from global developmental delay in early childhood, to no current academic support, to severe intellectual disability. More than half of participants had at least one health condition in the Allergic; Cardiac; Dental/Palate/Jaw; and Ear, Nose, and Throat categories. Age at molecular WSD diagnosis ranged from three weeks to 13 years, with a median age of 3.5 months. Eleven children received a molecular diagnosis of WSD by six months of age.

From the semi-structured interviews, we conceptualized five overarching themes: (1) uncertainty has a profound emotional impact on caregivers, (2) while the prospect of lifelong caregiving is stressful, caring for a child with WSD is rewarding, (3) partnerships between healthcare providers and families are critical, (4) three key areas for assessment in future WSD clinical studies include aniridia/vision, behavioral/psychiatric, and speech/communication conditions, and (5) the WSD community is a powerful support system.

### Theme 1: Uncertainty has a profound emotional impact on caregivers

In all interviews, the emotional toll of uncertainty was a salient point. Caregivers expressed distress over: (1) the limited information about WSD provided at the time of diagnosis, (2) the unknowns about the future health and well-being of their child with WSD, and (3) the lack of a long-term care plan for their child once a caregiver is gone or otherwise unable to provide care.

#### Uncertainty at the time of diagnosis

Among caregivers who knew their child’s early health history, most shared that early identification of aniridia led to an early molecular diagnosis of WSD. Despite the certainty of a molecular diagnosis, caregivers consistently reported that the time of diagnosis was filled with uncertainty. Several participants noted their child’s healthcare providers delivered only a limited overview of WSD at the time of diagnosis:

We went and saw a neurologist, and we discussed it, with the most important part obviously being that every three months we’d need to get a kidney ultrasound because [our child] was at such a high risk of Wilms. But everything else, there was just a lot more unknown than known. But that likely [our child] would have significant visual issues and probably developmental delays and such. There wasn’t really much more. (Participant 1)

With limited medical management information from their child’s care team, some caregivers were unsure how to manage aniridia. Participant 2 stated:

They gave you your kid and say, “Take [them] home and love [them].” And I was like, “That’s the only thing I know how to do.” But you need to tell me what to do with this child. Could [they] go outside? Could [they] be in a house with lights? We lived in the dark for a few weeks because we did not know if we were harming [their] eyes…. We all laugh about it in our support group, but I was looking for aniridia light bulbs.

Regarding ocular management, several caregivers shared that their child’s healthcare providers had limited knowledge of aniridia management. During an early hospitalization, Participant 9 noted, “I didn’t feel like the doctors really knew very much. They had the room completely black because they thought [my child] couldn’t see light.” Other caregivers dealt with uncertainty from their child’s care team upon identification of aniridia during surgery for another ophthalmic condition. During an early cataract surgery for their child, Participant 7 recalled, “Our eye doctor…said, ‘I’m in the middle of surgery. And I just realized, [your child] has aniridia, and I’m not supposed to be doing surgery on anyone with aniridia. But I can’t stop. What should I do?’”.

#### Uncertainty about the future health of children with WSD

Looking forward, the majority of participants voiced concerns about potential health complications of WSD:

It’s the anxiety of the what ifs. And I know that’s an anxiety for a lot of WAGR parents because it’s just uncertain. Wilms tumor treatment. Age of the oldest people who have WAGR are not that old. So life expectancy. What to expect? Technology. What could be? What won’t be? The anxiety is very hard to push down and away and live in that moment because so much could happen. (Participant 10)

Several caregivers also stated that they were concerned about the possibility of further vision loss and renal failure for their child. Thinking about vision, Participant 4 wondered if their child was “going to keep the vision [they have] now.” Elaborating on their fear of renal complications, Participant 10 stated, “Kidney failure scares the s*** out of me.” This participant specifically referenced stress around their child having only one kidney and being in stage 2 kidney failure, with the potential to develop another Wilms tumor.

Some caregivers discussed pressure around decision-making that could have long-lasting impacts on their child. Participant 13 referenced challenges associated with decisions related to their child’s “independence, health, and ability,” further noting “[Parents of children with WSD] are appointed responsibility for making decisions, and we spend a good amount of time wondering if we have chosen correctly.” Echoing this sentiment, Participant 5 described challenges of medical decision-making early in their child’s life with limited information. This participant expressed regret over proceeding with an early ophthalmic surgery for their child shortly after another surgery:

The retina surgery, which happened when [my child] was six months, was a very complex surgery. And I think that was the biggest mistake we made for [them]. We never went for second review, third review. The doctor said, “Let’s do a surgery.” We just agreed upon that. We think we should have waited…. At that time, we were not very sure what is good, what is bad…. That surgery was very bad for [my child] in terms of recoveries. [They] had bleeding from the eyes after the surgery, and [they were] crying so many nights…. It was a trauma for [them].

#### Uncertainty about who will care for an individual with WSD after their caregiver is unable to continue providing care

Regardless of their child’s age, most caregivers cited the uncertainty of who would provide care to their child with WSD in the future as a major source of stress. In particular, caregivers were concerned about experiencing a decline in their own health, passing away, or otherwise being unavailable to care for their child. In the short term, some caregivers shared they were unsure about available daycare options when their child with WSD reaches adulthood: “I don’t know what happens when [my child] is out of school. I’m guessing there’s day programs, but I don’t know. That’s a long way away, but it also feels really soon to try to figure out” (Participant 7). Other participants were concerned that the physical and emotional toll of caregiving may lead to difficulty providing future care, with Participant 11 noting “I’m afraid that I will be exhausted and will not be able to take care of [my child] because of burnout.” While some participants hoped that siblings or other loved ones would assume responsibility of future caregiving, others were hesitant to place this responsibility on family members:

Our biggest stress is at one point, we’re going to be gone. And the scariest thing in our life is who takes care of our kid. That’s a lot to put on [their sibling], and that’s not our expectation…. The clock moves really fast, and there’s ongoing guilt in place due to our inability to identify solutions. (Participant 1)

### Theme 2: While the prospect of lifelong caregiving is stressful, caring for a child with WSD is rewarding

While many participants discussed the exhaustion and stress associated with caregiving, they also emphasized the fulfilling nature of caring for a child with WSD. Participant 2 shared:

It’s so hard for my brain to wrap around the fact that I am going to be doing this until the day I die…. It already feels like we’ve been doing it so long…. But how does it make me feel? It’s an exhaustion that’s worth having…. I’m exhausted mentally, physically every day, but I’ll get up and do it every day because [my child] is worth it.

Reflecting on rewarding aspects of caregiving, several participants shared that their child with WSD has an incredible sense of humor. Participant 1 relayed, “I don’t think I’ve ever belly laughed so hard due to things [my child] has said or done.” Participant 2 added:

[My child] is nonverbal. So [their sibling]’s car broke down…We had to go pick [their sibling] up and call a tow truck…. [My child]’s obsessed with “Wheels on the Bus”…. When [their sibling] got in the car, [child with WSD] was playing the tow truck [version of] “Wheels on the Bus”…. So we all just looked at each other and started cracking up because [they] knew what we were doing. And [they were] basically teasing [their sibling] like “Haha your car broke down, and you have to wait for a tow truck.”

Some caregivers felt grateful for the innocence of their child with WSD, reporting that their child was largely unaffected by the negative emotions in the world. Participant 5 noted, “[My child] is such a pure soul. [They don’t] know anger, hatred, jealousy…. [Children with WSD] are just in this journey, and I feel we are blessed to support them.” Participant 7 shared a similar sentiment, discussing how having a child with WSD shifted their perspective of religion:

I turned away from God…. With [my child]’s diagnosis, it was really hard at first to understand why…. Recently, I had a personal breakthrough of realizing [my child] is exactly what I prayed for…. [They are] beyond that. [My child]’s living on a plain. [They’ll] never worry or be concerned for what people think of [them]. And that’s so beautiful. That’s God.

Many caregivers reported other positive worldview shifts after having a child with WSD, with an increased appreciation for “the little things.” Participant 12 discussed how their child with WSD enhanced their understanding of love: “[Our child] has this unbelievable love for people. [They love] everybody and give hugs to everybody. [They say] ‘hi’ to everyone with this huge smile…. The love that [our child] taught us is different love.”

### Theme 3: Partnerships between healthcare providers and families are critical

With the burden of numerous medical appointments, caregivers consistently expressed the need for their child’s healthcare providers to research WSD and become familiar with their child’s medical history prior to appointments. Participant 12 shared:

We…had a doctor that was proactive…and setting us up with multiple doctors from the beginning. That’s when we had a hard time because besides him, nobody else knew. And then we had to explain why we are there, why we are that early, and what we are looking for. So it’s us trying to process not knowing what to expect in the future and now looking for somebody to help, and they don’t know. And it’s frustrating. As a doctor, you should be trying to look it up and not just like, “Oh, I don’t know what it is.”

Participant 9 discussed the added stress of reciting their child’s medical history at each appointment, noting that having “good doctors” does not “make it any less stressful” when the doctors ask multiple questions about information in the chart. This participant added that these experiences can discourage families from engaging with medical care. In contrast, Participant 6 highlighted the positive impact of switching to a new care team that provided thorough, compassionate medical care: “We’re very fortunate. Our doctors listen to us and read the documentation that we give them. I have given the [recordings of WSD] conferences to our doctors…and they will look at those videos.” To provide a high level of care to children with WSD, another participant emphasized the importance of healthcare providers and families working as a team:

I did give [physician] some information, and she was not pleased at all. And she rescheduled [my child’s] surgeries. And then she was out of town. And we never saw her again. So she didn’t appreciate it…. You want to give them information, but you also don’t want to step on their toes. And you don’t want them to feel like you know everything because we don’t know everything. But they also don’t know everything. Some specialists are very open, and they want to work together, but some just want…you to sit still and listen. (Participant 4)

### Theme 4: Three key areas for assessment in future WSD clinical studies include aniridia/vision, behavioral/psychiatric, and speech/communication conditions

When asked which therapeutic target, if any, would have the most impact on individuals with WSD, caregivers highlighted five health categories. Vision conditions, including aniridia, were the most frequently cited target of interest. Behavioral/psychiatric conditions and speech/communication conditions followed. One participant desired a treatment for renal conditions, while another desired a therapy that would improve gross motor development for their child who was not yet walking. The challenges associated with these conditions for individuals with WSD and their families are outlined below. Several caregivers described improvements in various aspects of their child’s health over time, providing helpful context for selected therapeutic targets of interest.

#### Aniridia and vision conditions

The impact of complex ophthalmic conditions on both children with WSD and their families was a key topic raised by caregivers. On a day-to-day basis, some caregivers referenced difficulties associated with multiple ophthalmic surgeries and eye drops. Participant 1 noted that their child has received up to 18 eye drops per day, describing the complexities of balancing eye drop administration with school. In the setting of low vision, a few caregivers referenced how parsing out the primary component of developmental or cognitive delay is more challenging:

It was hard to figure out what was motoric versus what was visual…. Like I don’t know if you’re not meeting this milestone because you can’t see the ball and so you don’t want to crawl to it or if it’s because you physically aren’t strong enough to do that yet. So it could be a combination of the two, or it could just be one factor or the other…. That’s constantly the conversation that we have. Is it a visual component, or does [my child] have dyslexia? (Participant 10)

Most participants further referenced struggling to understand what their child could see: “Some days, [my child] can pick up the smallest thing up from the floor and eat it. And some days, [they’ll] still bump into a bigger thing” (Participant 5). Further reflecting on the unique challenges associated with understanding visual acuity in children with WSD, a few participants reported that their child’s vision seemed to be improving despite medical exams to the contrary. Participant 9 noted, “[My child’s] eyes have gotten worse every time we go. But functionally, [they’ve] gotten better with [their] eyes…. Something will be across the room, and [they’ll] crawl over and get it, where sometimes I wouldn’t think [they] could see that.”

Other caregivers reflected that participating in events can be challenging with low vision. Participant 13 noted, “We went to [a show]. We always have to sit right in the front, and then I’m never sure what [my child] can see. I tend not to buy tickets for things like that because it’s frustrating for [my child].” Another participant expressed frustration that events have wheelchair accessible areas but “not one has programs for visually impaired that you don’t have to pay an arm and a leg for seats where they can see” (Participant 10).

A few participants shared that their child’s low vision appeared to have a greater impact on themselves than on their child. Participant 6 noted, “That is a me thing, the vision thing. [My child] doesn’t care…. The vision does not slow [them] down.” Participant 1 discussed the grief experienced by themself and their partner due to worsening of their child’s ophthalmic conditions:

This last retinal detachment has…really sent us into the stages of grief again because we’ve been really good at holding off on different surgeries, not trying anything because we wanted to keep [our child’s] eyes in as good of a condition as possible knowing that there’s some real medications that could come down the road to help. And I think we’ve missed that opportunity…. [Our child’s] eyes have gotten too worse too fast.

#### Behavioral and psychiatric conditions

For several caregivers, behavioral and psychiatric conditions were the most difficult aspects of WSD. Describing the most challenging symptoms to manage as a caregiver, Participant 12 referenced “the constant talking and demanding things,” noting “You can’t even hear your thoughts.” Participant 6 echoed, “We can deal with all that other stuff that comes up. It’s the psychological – the autism with that demand avoidance. That’s the hardest part right now because [our child] gets very combative.” This participant further noted that pathological demand avoidance was hard on their child, making it difficult to understand “how this world works” and to deal with “outside noise” (Participant 6).

Consistently, participants shared that overstimulation, gastrointestinal conditions, and changes in routine contributed to behavioral difficulties. Several caregivers emphasized that outings or social events needed to be short to minimize overstimulation and meltdowns, as many children with WSD “do not like noise or crowded places” (Participant 5). When meltdowns occur for a child with WSD, most caregivers reported having to stay home or immediately leave an event: “There’s definitely times where we have to cancel plans or just drop everything and leave…. It’s a certain pitch to [our child’s] cry…. I know there’s not going to be any talking [them] out of it” (Participant 7). Participant 1 discussed how both gastrointestinal concerns and disruptions in routine impact their child’s behavior:

The biggest challenges we deal with are if [our child] gets overtired or constipated, [they] will act out a little bit. And…if routines are changed…[they] can really overreact and get quite upset…. We went [out], and the ice cream stand was down…. It took me 10 minutes to get [them] out of there. I had [them] carried on my side like a bag, and [they were] screaming, groaning, kicking.

A couple caregivers experienced injuries as a result of behavioral challenges. Participant 4 recalled: “[My child] would just scratch [themselves] and scratch me. I would go to work, and people would ask me…if I worked in the garden or had a cat at home because my arms were just full of scratches and blood.” Several participants shared that behavioral therapies had a major impact on curbing difficult behaviors:

When [my child] was younger, we had a lot of meltdowns. Thank God…those are well-controlled now. It was awful for three years…. I don’t know how my marriage survived…. No one slept. You could hardly breathe without a 12-hour screamfest starting. We have PTSD from that time period…. ABA [applied behavioral analysis] saved our lives…. That’s when meltdowns stopped…. I don’t think any of us would have survived any longer. (Participant 2)

Unique to caregivers of teenagers with WSD, one primary concern included the impact of mental health conditions on their teen and family. One caregiver reported that their teen was both anxious and angry about their WSD diagnosis:

The first signs of behavior came because [my child] became more aware of being different from others. [My child] is very frustrated that [they have] this disability. [They find] it an injustice that…[their sibling(s) have] no disability. (Participant 11)

Participant 13 shared that learning about the various manifestations of WSD contributed to their child’s anxiety, noting “[My child] really worries that [they] might have a developmental disability. And sometimes I think [my child] works too hard to prove [themselves].”

In terms of other mental health concerns, Participant 14 shared that one of the hardest aspects of caregiving was dealing with their child’s mood swings. Participant 11 also reported an episode of psychosis that was difficult to manage, during which time their child “did not support two people around [them], [their sibling(s)], any noise, or any changes.” A few caregivers raised questions about the treatment of psychiatric conditions for individuals with WSD. Participant 12 shared, “We tried different medications, and we distinctly see changes [in] personality. [Our child] is not loving. [They’re] more subdued.” Participant 12 added, “With the psychological component, we haven’t found a good solution for [our child’s] anxieties, frustrations, and OCDs.”

#### Speech and communication conditions

Several caregivers identified communication as one of the most difficult aspects of managing WSD. Participant 7 shared that communication was of interest as a therapeutic target, stating it would be beneficial “if [my child] was able to talk to me just about [their] daily needs.” Similarly, Participant 5 noted that better understanding their child’s daily needs would have a substantial impact on their family’s quality of life:

[Being] unable to express the desire or feelings is the most common cause of tantrums because you’re not able to communicate what’s going on with [them]…. When [they are] in pain, [my child] is not able to say what’s bothering [them]. So I am hoping that one day [they are] able to express that, and then we can help [them] better.

Like Participant 5, Participant 2 shared that communication challenges not only limit their understanding of their child’s needs but contribute to behavioral challenges. When describing the rationale behind selecting speech as the most desired therapeutic target for WSD, Participant 2 noted:

Behavior, voice, speech, and eyes would be my top. I don’t say the cancer stuff because I feel like that’s already an area people are trying to explore, and a lot of people can relate to cancer more than they can the eye issues…. Behavior’s been so challenging, but speech would change our whole life…. I would do speech because I think speech would fix the behavior. If [my child] couldn’t see, [they] would still have a voice.

#### Maintenance of chronic conditions and improvements in sleep, feeding, and behavior

The majority of caregivers reported that their child’s health remained stable and that feeding and sleep difficulties improved over time. Some caregivers described additional progress in the behavioral domain. Participant 3 shared that their child with WSD “is on a pretty good health path,” and Participant 2 echoed, “We’re in maintenance with a lot of the health stuff.” Regarding caregiving for children with WSD who have limited speech, several participants shared that caring for their child has become easier: “Over time, we have understood that when [our child] is gassy and constipated, this is how [they cry]. [They stretch their] body like this. So we are getting to know [our child] better and manage [them] well” (Participant 5). Another participant described the positive impact of feeding progress and the completion of chemotherapy on their family’s life:

[My child’s] feeding has gotten so much better…. The trials and tribulations of having the NG-tube on a mischievous, high-pain-tolerance hellion was very challenging. And then obviously chemo was very challenging, having weekly infusions and lots of nausea. The amount of vomit and refeeding and laundry was exhausting…. It’s relatively easy now compared to some of the daily grind of the tube and the laundry…. Most days are good days. (Participant 7)

In the context of sleep, some caregivers shared that sleep was previously challenging, with disruptions throughout the night that led to exhaustion for the entire family. Participant 2 noted, “If we got two hours of sleep when [our child] was little, we were all lucky.” With time, most caregivers, including Participant 2, noted that their child now sleeps in their own room with minimal disruptions to their family members’ sleep. A few participants described notable improvements in their child’s behavior, with one describing discontinuation of medications for emotional regulation: “A bad day is where [my child] would be upset for whatever reason and gets angry. [They] have that sometimes now. Not as bad as a few years ago. It’s much, much better” (Participant 4).

### Theme 5: The WSD community is a powerful support system

Several caregivers emphasized that the support of the WSD community, including the IWSA, has improved not only the medical care of their child with WSD but their family’s well-being. Participant 10 shared, “Our IWSA team and group have been amazing in supporting the unknown and just being a sounding board. Because we were scared, and they helped calm my nerves a lot.” Participant 9 reflected that WSD community members facilitated connections to needed specialists and helped normalize the experiences of their family:

The geneticist was like, “You need to find the WAGR group because they’ll be more helpful than the doctors here.” And that’s how I figured a lot of stuff out the first couple months. I had no clue if I didn’t have them. They gave me really good insight into what specialists [my child] would need…. They made it seem like it wasn’t like some weird case…. They were like, “Yeah, we do that too.” So it wasn’t as foreign.

Other participants highlighted the positive impact of attending events with the WSD community, including the IWSA’s annual WAGR Weekend event:

When we finally got to [WAGR Weekend], our world opened up. And since then, WAGR’s not so bad. I mean, it’s bad. But it’s not as scary anymore. And we got…super great doctors that…had the resources to spend more time with us as a family with a special needs, very active child. (Participant 6)

## Discussion

This mixed methods study provides a comprehensive exploration of the lived experiences of caregivers to children with WSD, offering critical insight into the needs and values of the WSD community. To guide WSD-specific clinical management and therapeutic development, this study focused on the phenotypic variability associated with WSD, gaps in care for individuals with WSD, sources of support for families living with WSD, and therapies that are of the highest priority to WSD community members. Based on the themes identified in this study, we have synthesized key clinical guidance for healthcare providers to improve the standard of care and support for families living with WSD (Table 3).

**Table 3:** Clinical guidance for healthcare providers: Supporting individuals with WSD and their families

Clinical Domain	Key Challenges & Caregiver Perspectives from Study Findings	Recommended Actions for Healthcare Providers
Diagnosis & Initial Counseling	• Uncertainty and limited information provided at diagnosis. • Lack of specific guidance on aniridia management (e.g., light sensitivity). • Feelings of unpreparedness among caregivers.	• Provide structured, comprehensive counseling beyond Wilms tumor surveillance. • Provide an overview of the spectrum of possible features using recent guidelines [1]. • Offer connections to resources, such as the International WAGR Syndrome Association (IWSA).
Ongoing Medical Care & Communication	• Burden of reciting child’s history at every appointment. • Dismissal of caregiver expertise and concerns. • Lack of provider knowledge of WSD.	• Review the patient’s chart *before* the appointment. • Acknowledge caregivers as essential partners in the care team. • Review WSD practice guidelines and common multi-systemic features [1].
Management of Key Symptom Domains	• Behavioral/Psychiatric: Often, the most challenging aspect. Association with overstimulation, GI issues, and disruptions in routine. • Vision: Difficulty distinguishing vision challenges and developmental/cognitive delay. Grief over progressive vision loss. • Communication: Frustration and tantrums due to inability to express needs/pain.	• Proactively screen for and manage behavioral challenges. Facilitate referrals to ABA, psychiatry, and behavioral therapies. • Acknowledge the impact of low vision and refer to developmental vision specialists. • Prioritize speech and augmentative/ alternative communication (AAC) interventions early and consistently.
Psychosocial & Future Planning	• Anxiety about future health (e.g., renal failure, vision loss). • Stress about long-term care plans. • Caregiver burnout and exhaustion.	• Initiate early, ongoing conversations about transition to adult care and future planning (e.g., financial, residential). • Routinely check in on caregiver mental health and provide referrals to mental health counseling or support groups. • Validate the emotional toll of WSD.
Community Resources	• The WSD community as a powerful source of practical advice and emotional support.	• Refer families to the IWSA or other support groups.*

*International WAGR Syndrome Association: www.wagr.org

### Broad phenotypic spectrum of WSD

With a wide range of reported health conditions spanning multiple organ systems, children with WSD in the present study reflected the broad phenotypic spectrum of WSD described in a recent cohort of more than 90 individuals with WSD [1]. Both classic and emerging features of WSD were represented. In the context of classic features, aniridia was the most common health condition for children with WSD in this study, aligning with previous WSD cohort studies. The frequency of Wilms tumor and/or nephrogenic rests in the present study was also consistent with the approximate 50% risk reported for the WSD population [1–3]. In terms of other genitourinary anomalies, the frequency of approximately 60% in the present WSD cohort aligned with that of other cohort reports [1]. The “range of developmental delay” category is where the present WSD cohort differed from prior cohort studies. Caregivers reported a higher rate of global developmental delay and intellectual disability among children with WSD in this study compared to the rate of approximately half in other WSD cohorts [1, 2, 16]. Similarly, a diagnosis of autism spectrum disorder was more common in this population compared to previous studies [1, 2, 16]. Outside the classic features of WSD, there was representation of symptoms across multiple other organ systems. One health condition category that predominated was gastrointestinal and feeding concerns, with 92% of children with WSD in this cohort having at least one gastrointestinal or feeding condition, echoing the high proportion in another WSD study [1].

Overall, reported health conditions of children with WSD in this cohort were largely consistent with those observed in previous cohort studies. Collection of paired quantitative clinical data and qualitative family-reported experiences offers insight into clinical features that families find most emotionally taxing, thereby informing WSD-specific clinical management, therapeutic development, and psychosocial support.

### Holistic WSD clinical management

Caregivers in this study demonstrated an incredible commitment to the health and well-being of their children with WSD. In addition to managing the emotional and physical demands of daily caregiving, caregivers exhibited relentless advocacy for their children, particularly within the healthcare space. The majority of caregivers highlighted opportunities for healthcare providers to enhance both the clinical care of their children with WSD and support of their family.

Insufficient provider preparation related to WSD education and medical chart review along with dismissal of WSD families’ concerns contributed to caregiver burnout and are deterrents to seeking medical care. Within the rare disease community, individuals and families consistently report an exhausting onus to serve as “experts” in their condition and to educate clinicians [17]. In view of the complex and potentially life-threatening medical conditions associated with WSD, it is essential that providers of children with WSD become familiar with both the medical history of their patients with WSD and WSD practice guidelines [1, 3]. Furthermore, care teams are encouraged to address the psychosocial needs of families at appointments. As behavioral and psychiatric conditions were reported as some of the most challenging conditions associated with WSD, providers across specialties are advised to check in with families about behavioral concerns and facilitate connections to supportive therapies.

Another key point raised by caregivers to children with WSD includes the challenges of navigating the unknown. Within the rare disease space, both the uncertainty of non-diagnostic genetic testing and the diagnostic odyssey garner a great deal of attention. On average, families spend over six years searching for a diagnosis [18]. For children with WSD in the present cohort, diagnosis occurred much earlier, often due to early identification of aniridia. While the uncertainty associated with an undiagnosed medical condition can induce a high level of stress for families, the uncertainty of the future after a rare disease diagnosis can be equally stressful. Following a WSD diagnosis, WSD families were incredibly worried about what came next for their child and family. To further enhance the psychosocial support of WSD families, it is recommended that healthcare teams inform families about WSD community supports like the IWSA. As noted in the WSD practice guidelines, providers can also consider offering referrals to mental health providers for enhanced psychosocial support of caregivers, especially during the time of WSD diagnosis [1]. Across ages of children with WSD, caregivers expressed concern over future care placements for their children, referencing both day programs and adult residential programs. While not all individuals with WSD will require this level of support, healthcare teams for children with WSD are encouraged to start an early dialogue with families around transitioning to adult care and available resources, such as those related to financial planning.

Taken together, healthcare providers are strongly advised to review WSD practice guidelines, become familiar with the medical history of their patients with WSD, and provide psychosocial support to families. Psychosocial support can include check-ins about behavioral concerns and caregiver well-being, referrals to mental health providers and WSD community supports, as well as resources around transitioning to adult care.

### Tailored WSD therapeutic development

As rare disease communities increasingly look to precision medicine, it is critical to center therapeutic development in the needs of these communities. Prior DCMs have indirectly identified therapeutic targets of importance to families living with rare genetic conditions, wherein the most frequently referenced health concepts in interviews with families are equated with therapeutic targets of highest priority [7–11]. In contrast, we directly asked caregivers of children with WSD which treatment targets, if any, were of greatest importance. Based on the findings of this study, future clinical research on WSD should consider including clinical endpoints around aniridia and vision improvements, emotional and behavioral regulation, as well as enhanced communication to improve the lives of both individuals with WSD and their caregivers. Clinical researchers may also consider renal function and gross motor development improvements as clinical endpoints. Given the early diagnoses of many individuals with WSD, advances in WSD-specific therapies have the potential to substantially impact the progression of WSD symptoms.

Despite WSD’s rare prevalence, estimated as under 1 in 500,000, therapies developed for WSD have the potential to benefit more common conditions with overlapping clinical features and molecular etiologies. For example, aniridia is much more common than WSD, with a prevalence of around 1:40,000 to 1:100,000 [19]. Most often, aniridia without WSD is caused by deletions or other variants in the *PAX6* gene [20, 21]. With the overlap of *PAX6* involvement and aniridia, development of a *PAX6*-targeted therapy could treat both WSD and *PAX6*-related aniridia.

### Limitations

When interpreting the results of this study, several limitations should be considered. First, our study was based on caregiver-reported data rather than data reported by individuals with WSD. The needs and priorities of individuals living with WSD may be different than those of their caregivers. Second, the study focused on children and adolescents; thus, the challenges faced by adults with WSD and their caregivers are not represented. Additionally, the limited racial and gender diversity among caregivers restricts the generalizability of our findings across different racial/ethnic groups and gender identities. WSD has been observed in populations worldwide; however, individuals of European descent are most frequently represented in the literature. This discrepancy may reflect social determinants of health that contribute to disparities in genetic diagnosis and clinical care for underrepresented minority groups. Further research is needed to address these gaps. Participants were also recruited from one institution and one patient advocacy organization, potentially influencing the reported experiences and perspectives of the WSD community. This recruitment strategy may have selected families who are more resource-connected and highly engaged with medical and community support systems, potentially overlooking the perspectives of more isolated families. Furthermore, children with WSD described in the present study had higher levels of developmental delay and intellectual disability compared to other WSD cohorts, which may limit the generalizability of our findings to the broader WSD community. Finally, as with any qualitative study, findings may be subject to recall and social desirability bias.

### Future directions

Our research highlights the need for additional studies that capture the experiences of adults with WSD and their caregivers and focus on chronic health conditions, mental health differences in adulthood, and life expectancy of individuals with WSD. Research on facilitators and barriers for transitioning to adult care would further complement this work. Future iterations of this work could include individuals with WSD, families outside the United States and Europe, and individuals who speak languages other than English.

## Conclusions

This work describes the broad phenotypic variability of WSD and associated impacts on children with WSD and their caregivers. Notably, our study identified therapeutic targets desired by the WSD community, which should be considered when developing clinical trial endpoints for future WSD clinical studies. Moreover, our findings underscore the need for holistic, compassionate care that addresses the emotional toll of key features of WSD, particularly vision, communication, and behavioral challenges. Through awareness of the psychosocial impact of WSD on caregivers, providers can be more prepared to refer families to appropriate resources, including patient advocacy groups. Taken together, this study contributes to a more comprehensive understanding of WSD, paving the way for improved patient care, community support, and targeted interventions.

## Supplementary Information

Below is the link to the electronic supplementary material.

Supplementary Material 1

Supplementary Material 2

Supplementary Material 3

## Data Availability

The datasets generated and/or analyzed during the current study are not publicly available due to the risk of identifying participants.

## References

[1] Duffy KA, Trout KL, Gunckle JM, Krantz SM, Morris J, Kalish JM. Results from the WAGR Syndrome Patient Registry: Characterization of WAGR spectrum and recommendations for care management. Front Pediatr. 2021;9:733018. 34970513 10.3389/fped.2021.733018 PMC8712693

[2] Fischbach BV, Trout KL, Lewis J, Luis CA, Sika M. WAGR syndrome: A clinical review of 54 cases. Pediatrics. 2005;116(4):984–8. 16199712 10.1542/peds.2004-0467

[3] Kalish JM, Becktell KD, Bougeard G, Brodeur GM, Diller LR, Doria AS, et al. Update on surveillance for Wilms tumor and hepatoblastoma in Beckwith–Wiedemann syndrome and other predisposition syndromes. Clin Cancer Res. 2024;30(23):5260–9. 39320341 10.1158/1078-0432.CCR-24-2100 PMC11611621

[4] Han JC, Liu QR, Jones M, Levinn RL, Menzie CM, Jefferson-George KS, et al. Brain-Derived Neurotrophic Factor and obesity in the WAGR syndrome. N Engl J Med. 2008;359(9):918–27. 18753648 10.1056/NEJMoa0801119 PMC2553704

[5] Sapio MR, Iadarola MJ, LaPaglia DM, Lehky T, Thurm AE, Danley KM, et al. Haploinsufficiency of the brain-derived neurotrophic factor gene is associated with reduced pain sensitivity. Pain. 2019;160(5):1070–81. 30855519 10.1097/j.pain.0000000000001485 PMC6476691

[6] U.S. Department of Health and Human Services FDA Center for Drug Evaluation and Research, U.S. Department of Health and Human Services FDA Center for Biologics Evaluation and Research, U.S. Department of Health and Human Services FDA Center for Devices and Radiological Health. Guidance for industry: Patient-reported outcome measures: Use in medical product development to support labeling claims: Draft guidance. Health Qual Life Outcomes. 2006;4(1):79. 17034633 10.1186/1477-7525-4-79 PMC1629006

[7] Nabbout R, Auvin S, Chiron C, Thiele E, Cross H, Scheffer IE, et al. Perception of impact of Dravet syndrome on children and caregivers in multiple countries: Looking beyond seizures. Dev Med Child Neuro. 2019;61(10):1229–36. 10.1111/dmcn.14186 30828793

[8] Schaare D, Allison K, Skorge K, Fox P, Lusk L, Sarasua SM, et al. Living with CACNA1A-related hemiplegic migraine, a disease concept model. Front Neurol. 2024;15:1460187. 39555480 10.3389/fneur.2024.1460187 PMC11565606

[9] Sullivan KR, Ruggiero SM, Xian J, Thalwitzer KM, Ali R, Stewart S, et al. A disease concept model for *STXBP1*-related disorders. Epilepsia Open. 2023;8(2):320–33. 36625631 10.1002/epi4.12688 PMC10235567

[10] WillgossT, Cassater D, Connor S, Krishnan ML, Miller MT, Dias-Barbosa C, et al. Measuring what matters to individuals with Angelman syndrome and their families: Development of a patient-centered disease concept model. Child Psychiatry Hum Dev. 2021;52(4):654–68. 32880036 10.1007/s10578-020-01051-z PMC8238699

[11] Connors KL, Carmichael NE, Bichell TJ, Dies KA, Frazier ZJ. Development of a patient and caregiver-centered pediatric disease concept model for Kleefstra syndrome. Sage Open Pediatr. 2025;12:30502225251336880. 40612157 10.1177/30502225251336880 PMC12220866

[12] Malterud K, Siersma VD, Guassora AD. Sample size in qualitative interview studies: Guided by information power. Qual Health Res. 2016;26(13):1753–60. 26613970 10.1177/1049732315617444

[13] Braun V, Clarke V. Using thematic analysis in psychology. Qualitative Res Psychol. 2006;3(2):77–101.

[14] Braun V, Clarke V. Can I use TA? Should I use TA? Should I not use TA? Comparing reflexive thematic analysis and other pattern-based qualitative analytic approaches. Couns Psychother Res. 2021;21(1):37–47.

[15] Maxwell JA. A realist approach for qualitative research. Thousand Oaks: SAGE; 2012. p. 222.

[16] Xu S, Han JC, Morales A, Menzie CM, Williams K, Fan YS. Characterization of 11p14-p12 deletion in WAGR syndrome by array CGH for identifying genes contributing to mental retardation and autism. Cytogenet Genome Res. 2008;122(2):181–7. 19096215 10.1159/000172086

[17] Carmichael N, Tsipis J, Windmueller G, Mandel L, Estrella E. Is it going to hurt? The impact of the diagnostic odyssey on children and their families. J Genet Couns. 2015;24(2):325–35. 25277096 10.1007/s10897-014-9773-9

[18] Benito-Lozano J, López-Villalba B, Arias-Merino G, Posada de la Paz M, Alonso-Ferreira V. Diagnostic delay in rare diseases: Data from the Spanish rare diseases patient registry. Orphanet J Rare Dis. 2022;17(1):418. 36397119 10.1186/s13023-022-02530-3 PMC9670379

[19] Nelson LB, Spaeth GL, Nowinski TS, Margo CE, Jackson L. Aniridia. A review. Surv Ophthalmol. 1984;28(6):621–42. 6330922 10.1016/0039-6257(84)90184-x

[20] Grønskov K, Olsen JH, Sand A, Pedersen W, Carlsen N, Jylling A, et al. Population-based risk estimates of Wilms tumor in sporadic aniridia. Hum Genet. 2001;109(1):11–8. 11479730 10.1007/s004390100529

[21] Robinson DO, Howarth RJ, Williamson KA, van Heyningen V, Beal SJ, Crolla JA. Genetic analysis of chromosome 11p13 and the *PAX6* gene in a series of 125 cases referred with aniridia. Am J Med Genet. 2008;146A(5):558–69. 10.1002/ajmg.a.32209 18241071

