# Supplementary material for: A mixed methods study of the impact of WAGR spectrum disorder on individuals and their caregivers
Source: Orphanet J Rare Dis. 2026 Apr 24;21:221. doi: 10.1186/s13023-026-04360-z (PMC13276935; doi:10.1186/s13023-026-04360-z)
Supplement: Supplementary file 1 — Supplementary Material 1 [file 13023_2026_4360_MOESM1_ESM.pdf]

Appendix 1: Survey

Caregiver Demographic Information

What is your age in years?

____

What sex were you assigned at birth (on your original birth certificate)?

- ◯ Female
- ◯ Male
- ◯ Unsure
- ◯ Not listed. Please describe in the below text box
- ◯ Prefer not to answer

Sex assigned at birth:

____

What is your current gender identity? Please select all that apply.

- ☐ Woman
- ☐ Man
- ☐ Transgender or of trans experience
- ☐ Non-binary
- ☐ Genderqueer
- ☐ Genderfluid
- ☐ Agender
- ☐ Questioning
- ☐ Not listed. Please describe in the below text box
- ☐ Prefer not to answer

Current gender identity:

____

How would you describe your racial/ethnic identity? Please select all that apply.

- ☐ Black, African American, or African
- ☐ American Indian or Alaska Native
- ☐ Asian
- ☐ Middle Eastern or North African
- ☐ Native Hawaiian or other Pacific Islander
- ☐ Hispanic, Latino, or Spanish
- ☐ White
- ☐ None of these fully describe me. Please describe in the below text box
- ☐ Prefer not to answer

Racial/ethnic identity:

____

What is your employment status?

- ◯ Full-time
- ◯ Part-time
- ◯ Not currently working outside the home
- ◯ Prefer not to answer

What is the highest level of education that you completed?	◯ Less than a high school degree<br>◯ High school diploma/GED<br>◯ Vocational/professional certificate<br>◯ Some college<br>◯ Associate's degree<br>◯ Bachelor's degree<br>◯ Master's or graduate degree<br>◯ Doctoral or other professional degree<br>◯ Not listed. Please describe in the below text box
Highest education level:	________________________
How many children are living in your household (including your loved one with WAGR)?	________________________
What is your relationship to your loved one with WAGR?	◯ Parent<br>◯ Relative<br>◯ Not listed<br>◯ Prefer not to answer
Please specify type of parent.	◯ Biological parent<br>◯ Adoptive parent<br>◯ Stepparent<br>◯ Foster parent<br>◯ Not listed<br>◯ Prefer not to answer
Please specify.	________________________
Please specify relationship.	________________________
Please specify relationship.	________________________
	______________________________

Demographic Information for the Individual with WAGR Spectrum Disorder

What is the name of your loved one with WAGR?	________________________
What is the age of your loved one with WAGR (in years)?	________________________
What sex was your loved one with WAGR assigned at birth (on the original birth certificate)?	◯ Female<br>◯ Male<br>◯ Unsure<br>◯ Not listed. Please describe in the below text box<br>◯ Prefer not to answer

Sex assigned at birth: __________________________________

What is your loved one with WAGR's current gender identity? Please select all that apply.

- ☐ Woman/girl
- ☐ Man/boy
- ☐ Transgender or of trans experience
- ☐ Non-binary
- ☐ Genderqueer
- ☐ Genderfluid
- ☐ Agender
- ☐ Questioning
- ☐ Not listed. Please describe in the below text box
- ☐ Prefer not to answer

Current gender identity: ____________________________

How would you describe the racial/ethnic identity of your loved one with WAGR? Please select all that apply.

- ☐ Black, African American, or African
- ☐ American Indian or Alaska Native
- ☐ Asian
- ☐ Middle Eastern or North African
- ☐ Native Hawaiian or other Pacific Islander
- ☐ Hispanic, Latino, or Spanish
- ☐ White
- ☐ None of these fully describe my loved one with WAGR. Please describe in the below text box
- ☐ Prefer not to answer

Racial/ethnic identity: ____________________________

____

Birth History

Please fill in the birth weight of your loved one with WAGR. Please specify units (pounds, ounces, or grams). ____________________________

Please fill in the birth length of your loved one with WAGR. Please specify units (inches or centimeters). ____________________________

Please fill in the gestational age at birth (in weeks) of your loved one with WAGR. ____________________________

Were there any complications during delivery or within the first month after birth for your loved one with WAGR?

- ◯ Yes
- ◯ No
- ◯ Unsure

Please describe the delivery and/or birth complications. __________________________________

____

Eye/Vision Conditions

Either currently or in the past, has your loved one with WAGR had: structural differences in the eye(s) or vision loss?

- ◯ Yes
- ◯ No
- ◯ Unsure

Please select all that apply.

- ☐ Aniridia (partial or complete absence of the iris)
- ☐ Aniridia fibrosis syndrome
- ☐ Nystagmus (rapid, uncontrollable eye movements)
- ☐ Cataract(s)
- ☐ Glaucoma
- ☐ Optic nerve hypoplasia (small or underdeveloped optic nerve)
- ☐ Foveal/macular hypoplasia (underdevelopment of the fovea)
- ☐ Amblyopia (lazy eye)
- ☐ Strabismus (crossing of the eye(s))
- ☐ Corneal keratopathy/pannus (growth of blood vessels on the cornea)
- ☐ Hyperopia (difficulty seeing close up)
- ☐ Myopia (difficulty seeing far)
- ☐ Peter's Anomaly
- ☐ Retinal detachment
- ☐ Aphakia (missing lens in the eye that occurs, for example, as the result of cataract surgery)
- ☐ Coloboma (missing tissue in part of the eye)
- ☐ Anisocoria (different sized pupils)
- ☐ Heterochromia (different colored eyes)
- ☐ Microcornea (small cornea)
- ☐ Microphthalmos (very small eye(s))
- ☐ Anophthalmia (absence of one or both eyes)
- ☐ Other

Please specify "other."

____

____

Cardiac Conditions

Either currently or in the past, has your loved one with WAGR experienced problems with: blood pressure, cholesterol, the structure of the heart, or the function of the heart?

- ◯ Yes
- ◯ No
- ◯ Unsure

Please select all that apply.	☐ Hypertension (high blood pressure)<br>☐ Heart murmur<br>☐ Atrial septal defect (ASD)<br>☐ Patent foramen ovale (PFO)<br>☐ Ventricular septal defect (VSD)<br>☐ Coarctation of the aorta<br>☐ Interrupted aortic arch<br>☐ Patent ductus arteriosus (PDA)<br>☐ Pulmonary valve stenosis<br>☐ Pulmonary valve regurgitation<br>☐ Pulmonary atresia<br>☐ Mesocardia (heart in the middle of the chest)<br>☐ Hyperlipidemia (high cholesterol)<br>☐ Other
Please specify other heart or blood pressure conditions.	______________________
Did any of these heart differences require surgery?	◯ Yes<br>◯ No<br>◯ Unsure
Please specify which heart difference(s) required surgery:	______________________
	____________________________

Gastrointestinal and Feeding Conditions

Either currently or in the past, has your loved one with WAGR experienced problems with: feeding or with the gastrointestinal (GI) system (stomach, liver, pancreas, small intestine, large intestine/colon, esophagus, rectum, or anus)?	◯ Yes<br>◯ No<br>◯ Unsure

Please select all that apply.	☐ Feeding problems<br>☐ Pyloric stenosis (thickening of the pylorus muscle leading to projectile vomiting)<br>☐ Chronic vomiting<br>☐ Chronic constipation<br>☐ Chronic diarrhea<br>☐ Irritable bowel syndrome (IBS)<br>☐ Inflammatory bowel disease (IBD)<br>☐ Gastroesophageal reflux disease (GERD)<br>☐ Diaphragmatic hernia<br>☐ Inguinal hernia<br>☐ Umbilical hernia<br>☐ Intestinal malrotation<br>☐ Peptic ulcers (open sores in the duodenum (top of the small intestine) or in the stomach lining)<br>☐ Gastroparesis (slow movement of food through the stomach)<br>☐ Pancreatitis (inflammation of the pancreas)<br>☐ Gallstones<br>☐ Gallbladder removal (cholecystectomy)<br>☐ Anal stenosis (narrowing of the anal canal)<br>☐ Anorectal malformation (birth differences of the anus or rectum that interfere with passing stool/bowel movements)<br>☐ Other
Please select all that apply.	☐ Nasogastric tube (NG tube)<br>☐ Gastrostomy tube (G tube)<br>☐ Dysphagia (difficulty swallowing)<br>☐ Choking with feeds<br>☐ Difficulty latching<br>☐ Hyperphagia (extreme hunger leading to overeating)<br>☐ Other
Please specify other feeding problems.	__________________________
Please specify other GI conditions.	__________________________
	__________________________________

Ear, Nose, and Throat Conditions

Either currently or in the past, has your loved one with WAGR had structural differences of, problems with, or surgeries on: the ear(s), nose, or throat?	○ Yes<br>○ No<br>○ Unsure
Please select all that apply.	☐ Ear differences present at birth<br>☐ Hearing loss/Deafness<br>☐ Anosmia (partial or full loss of smell)<br>☐ Obstructive Sleep Apnea (OSA)<br>☐ Tonsillectomy (surgery that removes the tonsils)<br>☐ Adenoidectomy (surgery that removes the adenoids)<br>☐ Tympanostomy tube placement (ear tube insertion)<br>☐ Other

Please specify "ear differences" (e.g., low-set ears; atypical shape or fold of the outside portion of the ear (pinna/auricle)).	__________________________
Is the hearing loss on both sides or one side?	◯ Both sides (bilateral)<br>◯ One side (unilateral)<br>◯ Unsure
Is the hearing loss conductive or sensorineural?	◯ Conductive<br>◯ Sensorineural<br>◯ Both conductive and sensorineural<br>◯ Unsure
Does your loved one with WAGR use any devices to help them hear?	◯ Yes<br>◯ No
Please select all that apply.	☐ Cochlear implant(s)<br>☐ Hearing aid(s)<br>☐ Other
Please specify other hearing device.	__________________________
Please specify other ear, nose, or throat conditions.	__________________________
	________________________________

Dental/Palate/Jaw Conditions

Either currently or in the past, has a dentist or other healthcare provider noted that your loved one with WAGR has: differences in their teeth (like differences in the size, shape, color, amount, or location of the teeth) or in their mouth (like differences in the palate or jaw)?	◯ Yes<br>◯ No<br>◯ Unsure
Please select all that apply.	☐ Dental cavity/cavities<br>☐ Missing teeth<br>☐ Extra teeth<br>☐ Crowded teeth<br>☐ Widely spaced teeth<br>☐ Large gap between top two front teeth<br>☐ Large teeth<br>☐ Tooth in palate/roof of mouth<br>☐ Narrow palate<br>☐ High-arched palate<br>☐ Cleft palate<br>☐ Cleft lip<br>☐ Enamel hypoplasia (missing or absent tooth enamel)<br>☐ Weak/soft tooth enamel<br>☐ Malocclusion (underbite or overbite)<br>☐ Other
Please specify "other."	__________________________

Conditions involving the Extremities

Either currently or in the past, has your loved one with WAGR experienced differences in the structure or size of: the hands, feet, legs, arms, or nails?

◯ Yes
◯ No
◯ Unsure

Please select all that apply.

- ☐ Lateralized overgrowth/hemihypertrophy/hemihyperplasia (overgrowth of one side or one part of the body)
- ☐ Small hands/fingers for age
- ☐ Syndactyly (fused/webbed fingers or toes)
- ☐ Polydactyly (extra fingers or toes)
- ☐ Fifth finger clinodactyly (curved, short fifth finger)
- ☐ Small feet/toes for age
- ☐ Flat feet
- ☐ Club foot
- ☐ Toe-walking
- ☐ In-toeing (turning in of feet while walking)
- ☐ Recurrent ingrown toenails
- ☐ Small nails for age
- ☐ Dysplastic (atypical) nails
- ☐ Contractures of fingers/toes (unable to fully open the fingers or toes)
- ☐ Other

Please specify "other."

____

Musculoskeletal Conditions

Either currently or in the past, has your loved one with WAGR experienced problems with: the bones or muscles?

◯ Yes
◯ No
◯ Unsure

Please select all that apply.

- ☐ Scoliosis (curvature of the spine)
- ☐ Multiple hereditary exostoses (MHE) (benign bone tumors)
- ☐ Osteopenia (moderately decreased bone density)
- ☐ Osteoporosis (severely decreased bone density that can lead to fractures)
- ☐ Fracture (broken bone)
- ☐ Hypotonia (low muscle tone)
- ☐ Hypertonia (high muscle tone)
- ☐ Other

Please specify "other."

____

____

Conditions involving Tumors, Cancer, and Polyps

Either currently or in the past, has your loved one with WAGR had: tumors, cancer, and/or colon polyps?
◯ Yes
◯ No
◯ Unsure

Please select all that apply.
☐ Wilms tumor
☐ Nephrogenic rests
☐ Colon polyps
☐ Skin cancer
☐ Gonadoblastoma
☐ Other

Has your loved one with WAGR received treatment for the tumor(s), cancer, and/or colon polyp(s)?
◯ Yes
◯ No
◯ Unsure

Please select all treatments that apply.
☐ Surgery
☐ Chemotherapy
☐ Radiation therapy
☐ Other

Please specify the other type of treatment.
____

Has your loved one with WAGR had more than one Wilms tumor?
◯ Yes
◯ No
◯ Unsure

At what age was the (first) Wilms tumor identified? Please specify months or years.
____

Where was the (first) Wilms tumor?
◯ Right kidney
◯ Left kidney
◯ Unsure

When the (first) Wilms tumor was identified, what was the stage of the tumor?
◯ Stage I
◯ Stage II
◯ Stage III
◯ Stage IV
◯ Stage V
◯ Unsure

Was the (first) Wilms tumor found on ultrasound screening?
◯ Yes
◯ No
◯ Unsure

What led to the identification of the (first) Wilms tumor?
____

At what age was the second Wilms tumor identified? Please specify months or years.
____

Where was the second Wilms tumor?	◯ Right kidney<br>◯ Left kidney<br>◯ Unsure
When the second Wilms tumor was identified, what was the stage of the tumor?	◯ Stage I<br>◯ Stage II<br>◯ Stage III<br>◯ Stage IV<br>◯ Stage V<br>◯ Unsure
Was the second Wilms tumor found on ultrasound screening?	◯ Yes<br>◯ No<br>◯ Unsure
What led to the identification of the second Wilms tumor?	________________________
Has your loved one with WAGR had more than two Wilms tumors?	◯ Yes<br>◯ No<br>◯ Unsure
For each additional Wilms tumor, please specify 1) the age that the Wilms tumor was identified (and specify months or years); 2) whether the Wilms tumor was in the right kidney or the left kidney; 3) the staging of the Wilms tumor; and 4) what led to the identification of the Wilms tumor.	______________________________
At what age were the colon polyps identified? Please specify months or years.	________________________
At what age was the skin cancer identified? Please specify months or years.	________________________
At what age was the gonadoblastoma identified? Please specify months or years.	________________________
Please specify the other type(s) of tumor(s), cancer, or polyp(s).	________________________
Please specify at what age the other type(s) was found (and specify months or years).	________________________

____

Genitourinary Conditions

Either currently or in the past, has your loved one with WAGR experienced problems with: the kidney(s), urinary tract, bladder, urine, or reproductive organs/genitalia?	◯ Yes<br>◯ No<br>◯ Unsure
Please select all that apply.	☐ Chronic kidney disease<br>☐ Polycystic / Cystic kidney<br>☐ Hypoplastic kidney (underdevelopment of the kidney)<br>☐ Duplicate kidney<br>☐ Horseshoe kidney<br>☐ Renal agenesis (absence of a kidney from the time of birth)<br>☐ Renal tissue disorganization<br>☐ Focal segmental glomerulosclerosis (FSGS) (development of scar tissue in the glomeruli, the kidney's filtering units)<br>☐ Hydronephrosis/dilated renal pelvis (swelling of one or both kidneys due to buildup of urine)<br>☐ Ureteric reflux / vesicoureteral reflux (VUR) (backward flow of urine from the bladder into the ureters and kidneys)<br>☐ Ureteral duplication (an extra ureter on one or both sides)<br>☐ More than three urinary tract infections (UTIs) per year<br>☐ Difficulty emptying the bladder<br>☐ Small bladder<br>☐ Large bladder<br>☐ Proteinuria (protein in the urine)<br>☐ Hematuria (blood in the urine)<br>☐ Ambiguous genitalia<br>☐ Differences in male/male-appearing reproductive organs/genitalia<br>☐ Differences in female/female-appearing reproductive organs/genitalia<br>☐ Other
Please specify the current stage of chronic kidney disease.	◯ Stage 1<br>◯ Stage 2<br>◯ Stage 3a<br>◯ Stage 3b<br>◯ Stage 4<br>◯ Stage 5 (kidney failure)<br>◯ Unsure
Please select all that apply.	☐ Cryptorchidism (undescended testicle(s))<br>☐ Hypospadias (opening of the penis on the underside rather than the tip)<br>☐ Micropenis<br>☐ Bifid scrotum<br>☐ Other
Please specify the additional male/male-appearing reproductive organ/genitalia difference(s).	__________________________________
Please select all that apply.	☐ Streak ovaries (underdeveloped ovaries)<br>☐ Bicornuate uterus (heart-shaped/divided uterus)<br>☐ Small or hypoplastic (underdeveloped) uterus<br>☐ Ovarian cysts / Polycystic ovarian syndrome (PCOS)<br>☐ Other

Please specify the additional female/female-appearing reproductive organ/genitalia difference(s). __________________________________

Please specify other genitourinary conditions. __________________________________

____

Endocrine Conditions

Either currently or in the past, has your loved one with WAGR experienced problems with: growth, weight, sugar levels, hormones, puberty, or the thyroid?

- ◯ Yes
- ◯ No
- ◯ Unsure

Please select all that apply.

- ☐ Short stature
- ☐ Tall stature
- ☐ Obesity
- ☐ Diabetes
- ☐ Prediabetes
- ☐ Hyperglycemia (high blood sugar)
- ☐ Hypoglycemia (low blood sugar)
- ☐ Precocious (early) puberty
- ☐ Delayed puberty
- ☐ Low testosterone
- ☐ Early menopause
- ☐ Hyperthyroidism (overactive thyroid)
- ☐ Hypothyroidism (underactive thyroid)
- ☐ Other

Please specify "other." __________________________________

Has your loved one with WAGR used growth hormone?

- ◯ Yes
- ◯ No
- ◯ Unsure

____

Respiratory Conditions

Either currently or in the past, has your loved one with WAGR experienced problems with: the lungs, breathing, or with respiratory illnesses?

- ◯ Yes
- ◯ No
- ◯ Unsure

Please select all that apply.

- ☐ Lung hypoplasia (underdevelopment of the lungs)
- ☐ Laryngomalacia (noisy breathing due to floppy tissue in the voice box)
- ☐ Tracheomalacia (noisy breathing due to floppy tissue below the voice box)
- ☐ Pharyngomalacia (collapse of the upper airway when breathing)
- ☐ Bronchomalacia (collapse of the lower airway when breathing)
- ☐ Bronchiectasis (widening/thickening of the airways (bronchi) from inflammation and infection)
- ☐ Chronic obstructive pulmonary disease (COPD)
- ☐ Reactive airway syndrome/pre-asthma
- ☐ Asthma
- ☐ Shallow breathing
- ☐ Shortness of breath
- ☐ Apnea (pauses in breathing, especially during sleep)
- ☐ Obstructive sleep apnea (OSA)
- ☐ Pneumonia
- ☐ Recurrent (more than twice per year) pneumonia
- ☐ Recurrent sinusitis
- ☐ More than seven colds per year
- ☐ Respiratory tract infections requiring antibiotics
- ☐ Other

Please specify "other."

____

____

Developmental, Learning, Psychiatric, Behavioral, and Emotional Conditions

Does your loved one with WAGR have a history of: developmental delay, intellectual disability, challenges with learning, autism spectrum disorder, ADHD, anxiety, depression, or other developmental/emotional/psychiatric/behavioral diagnoses?

- ◯ Yes
- ◯ No
- ◯ Unsure

Please select all conditions for which your loved one has a diagnosis.

- ☐ Global developmental delay
- ☐ Intellectual disability
- ☐ Learning disability in reading
- ☐ Learning disability in math
- ☐ Dyslexia
- ☐ Speech (expressive) delay
- ☐ Language (receptive) delay
- ☐ Auditory processing disorder
- ☐ Sensory integration disorder
- ☐ Social communication disorder
- ☐ Visual-perceptive disorder
- ☐ Visual motor deficit
- ☐ Autism spectrum disorder (ASD)
- ☐ Attention deficit/hyperactivity disorder (ADD/ADHD)
- ☐ Obsessive compulsive disorder (OCD)
- ☐ Oppositional defiance disorder (ODD)
- ☐ Anxiety
- ☐ Depression
- ☐ Panic attacks
- ☐ Bipolar disorder / manic depression
- ☐ Schizophrenia
- ☐ Pseudobulbar affect (neurological condition that causes outbursts of uncontrolled or inappropriate laughing or crying; these episodes don't match a person's internal emotional state)
- ☐ Other

Please specify "other."

____

____

Neurological Conditions

Either currently or in the past, has your loved one with WAGR had: seizures, migraines, structural differences of the brain, an atypical head size, an unusual response to pain, or differences in muscle tone?

- ○ Yes
- ○ No
- ○ Unsure

Please select all that apply.

- ☐ Epilepsy (seizures)
- ☐ Migraines
- ☐ Microcephaly (smaller than typical head size for your child's age)
- ☐ Macrocephaly (larger than the typical head size for your child's age)
- ☐ Agenesis of the corpus callosum (partial or complete absence of the area that connects the two sides of the brain)
- ☐ Intracranial hypertension / pseudotumor cerebri (buildup of pressure around the brain)
- ☐ Hypotonia (low muscle tone)
- ☐ Hypertonia (increased muscle tone)
- ☐ Spasticity (stiff or rigid muscles)
- ☐ Ataxia
- ☐ Insensitivity to pain / high pain tolerance
- ☐ Cerebral palsy
- ☐ Other

Please specify "other." ______________________________

____

Allergy Conditions

Either currently or in the past, has your loved one with WAGR had: allergies/allergic reactions?

- ◯ Yes
- ◯ No
- ◯ Unsure

Please select all that apply.

- ☐ Animal dander / dust allergy
- ☐ Hay fever/allergic rhinitis
- ☐ Latex / skin irritant allergy
- ☐ Atopic dermatitis/eczema
- ☐ Medication allergies
- ☐ Lactose intolerance
- ☐ Food allergies
- ☐ Other

Please specify "other." ______________________________

____

Other Conditions

Does your loved one with WAGR have a history of situs inversus (where the organs in the chest and abdomen are on the opposite side of the body)?

- ◯ Yes
- ◯ No
- ◯ Unsure

Please describe any other conditions that your loved one with WAGR is experiencing now or has experienced in the past that were not listed above. _____________________________________
