# Supplementary material for: A mixed methods study of the impact of WAGR spectrum disorder on individuals and their caregivers
Source: Orphanet J Rare Dis. 2026 Apr 24;21:221. doi: 10.1186/s13023-026-04360-z (PMC13276935; doi:10.1186/s13023-026-04360-z)
Supplement: Supplementary file 2 — Supplementary Material 2 [file 13023_2026_4360_MOESM2_ESM.docx]

**Supplemental File 2:** Interview guide

**Discussion about WAGR Spectrum Disorder**

1. **Background Information:** First, I would like to discuss some background information and the symptoms your child experiences because of WAGR spectrum disorder.
   1. *When was your child first diagnosed with WAGR spectrum and by whom?*
      - 1. *Age*
        2. *Neurologist*
        3. *PCP*
        4. *Geneticist*
        5. *Ophthalmologist*
        6. *Other*
        7. *Clinical vs molecular diagnosis*
   2. *What were the first symptoms that you noticed?*
      1. *Prompt on milestones if not mentioned*
         - 1. *Feeding difficulties*
           2. *Eye contact*
           3. *Rolling over*
           4. *Sitting*
           5. *Walking*
           6. *Talking*
   3. *What did a typical day look like when your child was a baby?*
2. **Typical Day:** Can you tell me what a typical day is like for the person with WAGR spectrum and your family now?
   1. *What is your normal routine?*
   2. *What are the daily challenges or difficulties that you face?*
   3. *What is the most difficult or challenging part of your typical day?*
   4. *How do you define a good day versus a bad day?*
   5. *What is the person with WAGR spectrum able to do on a ‘good’ day?*
   6. *What might make a ‘good’ day better than any other?*
   7. *What does a ‘bad’ day look like?*
   8. *What is the person with WAGR spectrum able to do/not able to do on a ‘bad’ day?*
   9. *What might make a ‘bad’ day worse than any other?*
   10. *Are there certain/specific activities that you or the person with WAGR spectrum is not able to participate in?*
   11. *Is there anything that you or the person with WAGR spectrum cannot do?*
3. **Typical Night:** Can you tell me what a typical night is like for the person with WAGR spectrum and your family now?
   1. *What is your normal routine?*
   2. *What are the nightly challenges or difficulties that you face?*
   3. *What is the most difficult or challenging part of your typical night?*
   4. *How do you define a good night versus a bad night?*
   5. *What is the person with WAGR spectrum’s sleep like on a ‘good’ night?*
   6. *What might make a ‘good’ night better than any other?*
   7. *What does a ‘bad’ night look like?*
   8. *What is the person with WAGR spectrum able to do/not able to do on a ‘bad’ night?*
   9. *What might make a ‘bad’ night worse than any other?*
4. **School/Work:** Does the person with WAGR spectrum attend school, work, or a daycare program?
   1. *How is the person with WAGR spectrum doing at school, work or a daycare program?*
   2. *Does the person with WAGR spectrum attend these programs regularly?*
   3. *What is their day like at school?*
   4. *Can you tell me about how the person with WAGR spectrum participates in school activities?*
5. **Socialization:** Can you tell me about the person with WAGR spectrum’s relationship with friends/family?
   1. *How does the person socialize?*
   2. *Does the person with WAGR spectrum socialize with children their own age?*
   3. *How does the person with WAGR spectrum socialize with friends outside of school?*
   4. *Thinking about aspects of your culture, faith, traditions, or community values, does having WAGR spectrum have any impact on your child’s ability to learn about, engage in, or align with these?*
6. **Community Interaction:** Tell me how the person with WAGR spectrum’s participation in the community is affected by WAGR spectrum.
   1. *What activities does the person find difficult to do because of WAGR spectrum?*
   2. *Is the person with WAGR spectrum able to participate in physical activities?*
7. **Communication:** Regarding communication - How do you know what the person with WAGR spectrum is feeling?
   1. *How do they let you know?*
8. **Behavior:** How does the person with WAGR spectrum act?
   1. *How does the person behave? At home? In public? Other places?*
9. **Emotions:** How does having WAGR spectrum make the person feel?
   1. *How do you know?*
10. **Accommodations:** Have you had to make any adjustments to your home or car to accommodate the person with WAGR spectrum?
    1. *What type of adjustments have you made?*
    2. *Have you had to pay for these adjustments yourself, or are you reimbursed? If reimbursed, by whom?*
11. **Meaningful Clinical Difference:** What changes in the person with WAGR spectrum’s health would make a big difference in their daily life?
    1. *What changes in the person’s health would make a big difference in your daily life?*
    2. *What changes in the person’s health would make a big difference in your family’s daily life?*
12. **Changes by Age:** When the person with WAGR spectrum was younger, were different symptoms challenging?
    1. *Have they changed over time?*
    2. *Can you please tell me the symptoms that were the most challenging when the person with WAGR spectrum was…*
       1. *5 years of age and younger?*
       2. *6-12 years?*
       3. *13-18 years?*
13. **Other:** Are there any other examples of how WAGR spectrum has affected your family’s life that we did not ask about and that you would like to mention?
14. **Most Difficult Symptoms:** l would like to understand which symptoms are most challenging to manage. Can you describe the three symptoms that your child is experiencing at this time that are the most challenging to manage as a parent/caregiver?
15. Tell me about what **THERAPIES** the person with WAGR spectrum receives or received in the past.
    1. *What impact did each therapy have on the person with WAGR spectrum’s life?*
    2. *How do you know?*
    3. *Have the THERAPIES the person receives changed over time? If so, how have they changed?*
16. Tell me about which types of **TREATMENT** the person with WAGR spectrum receives. Treatments might include dietary changes, medications, or other modifications to your child’s daily activities to improve or manage symptoms

**Parent/Caregiver Impact of WAGR spectrum disorder (15 minutes)**

1. **General:** What type of care do you provide for the person with WAGR spectrum?
   1. *Can you describe how caring for the person with WAGR spectrum makes you feel?*
   2. *What is the best thing about caring for the person with WAGR spectrum? Why?*
   3. *What is the hardest thing about caring for the person with WAGR spectrum? Why?*
2. **Typical Day:** Talk me through a typical day when you are caring for the person with WAGR spectrum. Tell me what it is like from the moment you wake up to the time you go to bed.
   1. *What kind of things can you do on a typical day?*
   2. *Is there anything you can’t do on a typical day?*
   3. *How do you feel physically on a typical day?*
   4. *How do you feel emotionally on a typical day?*
   5. *How has this changed since your child was diagnosed with WAGR spectrum?*
   6. *Does this change depend on what symptoms the person with WAGR spectrum is experiencing?*
3. **Good Day:** Describe a ‘good’ day when you are caring for the person with WAGR spectrum.
   1. *What kind of things can you do on a good day?*
   2. *Is there anything you can’t do on a good day?*
   3. *How do you feel physically on a good day?*
   4. *How do you feel emotionally on a good day?*
   5. *What makes a ‘good’ day better than any other day?*
   6. *How has this changed since your child was diagnosed with WAGR spectrum?*
4. **Bad Day:** Describe a ‘bad’ day when you are caring for the person with WAGR spectrum.
   1. *What kind of things are difficult on a bad day?*
   2. *Is there anything you can’t do on a bad day?*
   3. *How do you feel physically on a bad day?*
   4. *How do you feel emotionally on a bad day?*
   5. *What makes a ‘bad’ day worse than any other day?*
   6. *How has this changed since your child was diagnosed with WAGR spectrum?*
5. **Lifestyle Changes:** Tell me about things you find difficult or cannot do because of the person having WAGR spectrum.
   1. *Tell me about things you avoid doing because of the person having WAGR spectrum.*
6. **Concerns about the Individual with WAGR:** What concerns, if any, do you have about the person with WAGR spectrum?
   - 1. *You said [x] was a concern. Please explain further.*
        1. *How much are you concerned about this?*
        2. *How does this make you feel?*
7. **Concerns about Caregiver Abilities:** What concerns, if any, do you have about your ability to care for the person with WAGR spectrum?
   1. *You said [x] was a concern. Please explain further.*
      1. *How much are you concerned about this?*
      2. *How does this make you feel?*
   2. *What makes it difficult to care for the person with WAGR spectrum?*
      1. *How much is that a problem for you, if at all?*
   3. *What, if anything, makes caring for the person with WAGR spectrum easier?*
      1. *How does that make things easier?*
      2. *How does that make you feel?*
   4. *Do you feel you need assistance with caring for the person with WAGR spectrum? Why?*
      1. *What type of assistance do you need, for which type of caring activities, and how often?*
   5. *What, if anything, could make caring for the person with WAGR spectrum more difficult in the future?*
      1. *How might this make things more difficult?*
      2. *How might this make you feel?*
8. **Most Challenging Issues**: Of all the issues/problems that we talked about today, what three issues or problems are the most challenging/difficult today?
   1. *Why did you choose those three issues/problems?*
9. **Treatment Targets:** If you had to select one treatment target that would have the most impact on the life of the person with WAGR spectrum, what issue/problem would you choose as a treatment target (if anything)? What issue/problem, if treated, would have the most impact on your life as a caregiver?
   1. *Why did you choose this?*
10. **Other Studies:** Have you been part of other studies on WAGR spectrum; if so, which ones?
11. **Last Word:** Finally, is there anything else you would like to tell me about in relation to caring for the person with WAGR spectrum that we have not discussed?
    1. *Do you have any other concerns that have not been discussed today?*
