# Supplementary material for: A mixed methods study of the impact of WAGR spectrum disorder on individuals and their caregivers
Source: Orphanet J Rare Dis. 2026 Apr 24;21:221. doi: 10.1186/s13023-026-04360-z (PMC13276935; doi:10.1186/s13023-026-04360-z)
Supplement: Supplementary file 3 — Supplementary Material 3 [file 13023_2026_4360_MOESM3_ESM.docx]

**Supplemental File 3:** Health conditions of children with WSD

|  | | | | | | | |
| --- | --- | --- | --- | --- | --- | --- | --- |
| **Health category / condition** | | | | *n* | (%) | |  |
| Allergic | | | 8 | | (61.5) | |  |
|  | Atopic dermatitis/eczema | | 1 | | (7.7) |  |  |
|  | Food allergy | | 1 | | (7.7) |  |  |
|  | Latex allergy | | 1 | | (7.7) |  |  |
|  | Medication allergy | | 4 | | (30.8) |  |  |
|  | Seasonal allergies | | 2 | | (15.4) |  |  |
| Cardiac | | | 9 | | (69.2) | |  |
|  | Atrial septal defect | | 1 | | (7.7) | |  |
|  | Bicuspid aortic valve | | 1 | | (7.7) | |  |
|  | Coarctation of the aorta | | 1 | | (7.7) | |  |
|  | Heart murmur | | 2 | | (15.4) | |  |
|  | Hyperlipidemia | | 3 | | (23.1) | |  |
|  | Hypertension | | 5 | | (38.5) | |  |
| Dental, palate, and jaw | | | 9 | | (69.2) | |  |
|  | Cavities | | 1 | | (7.7) | |  |
|  | Crowded teeth | | 1 | | (7.7) | |  |
|  | Difficulty closing palate/stiff palate | | 1 | | (7.7) | |  |
|  | Discolored teeth | | 2 | | (15.4) | |  |
|  | Double tooth | | 1 | | (7.7) | |  |
|  | Enamel hypoplasia of adult teeth | | 1 | | (7.7) | |  |
|  | Extra teeth | | 1 | | (7.7) | |  |
|  | High-arched palate | | 3 | | (23.1) | |  |
|  | Malocclusion | | 2 | | (15.4) | |  |
|  | Small mouth | | 1 | | (7.7) | |  |
|  | | |  | |  | |  |
| **Health category / condition** | | | *n* | | (%) | |  |
| Developmental, cognitive, and psychiatric | | | 12 | | (92.3) | |  |
|  | Anxiety | | 3 | | (23.1) | |  |
|  | Attention-deficit/hyperactivity disorder | | 3 | | (23.1) | |  |
|  | Auditory processing disorder | | 1 | | (7.7) | |  |
|  | Autism spectrum disorder | | 7 | | (53.8) | |  |
|  | Depression | | 1 | | (7.7) | |  |
|  | Global developmental delay | | 12 | | (92.3) | |  |
|  | Intellectual disability | | 9 | | (69.2) | |  |
|  | Language delay | | 8 | | (61.5) | |  |
|  | Learning disability in math | | 5 | | (38.5) | |  |
|  | Learning disability in reading | | 5 | | (38.5) | |  |
|  | Obsessive-compulsive disorder | | 2 | | (15.4) | |  |
|  | Panic attacks | | 1 | | (7.7) | |  |
|  | Psychosis | | 1 | | (7.7) | |  |
|  | Sensory integration disorder | | 1 | | (7.7) | |  |
|  | Social communication disorder | | 1 | | (7.7) | |  |
|  | Speech delay | | 10 | | (76.9) | |  |
|  | Visual motor deficit | | 3 | | (23.1) | |  |
| Ear, nose, and throat | | | 7 | | (53.8) | |  |
|  | Adenoidectomy | | 6 | | (46.2) | |  |
|  | Ear asymmetry | | 1 | | (7.7) | |  |
|  | Hearing loss | | 2 | | (15.4) | |  |
|  | Low-set ears | | 2 | | (15.4) | |  |
|  | Obstructive sleep apnea | | 4 | | (30.8) | |  |
|  | Structural sinus differences | | 1 | | (7.7) | |  |
|  | Tonsillectomy | | 5 | | (38.5) | |  |
|  | Tympanostomy tube placement | | 5 | | (38.5) | |  |
| **Health category / condition** | | | | *n* | (%) | |  |
| Endocrine | | | | 5 | (38.5) | |  |
|  | Hyperglycemia | | 1 | | (7.7) | |  |
|  | Obesity | | 3 | | (23.1) | |  |
|  | Short stature | | 3 | | (23.1) | |  |
| Extremities | | | | 10 | (76.9) | |  |
|  | Brachydactyly | | 1 | | (7.7) | |  |
|  | Contractures | | 1 | | (7.7) | |  |
|  | Crooked toes | | 1 | | (7.7) | |  |
|  | Flat feet | | 1 | | (7.7) | |  |
|  | Hypoplastic nails | | 1 | | (7.7) | |  |
|  | Leg length discrepancy | | 1 | | (7.7) | |  |
|  | Overlapping toes | | 5 | | (38.5) | |  |
|  | Polydactyly | | 1 | | (7.7) | |  |
|  | Small hands/fingers for age | | 1 | | (7.7) | |  |
|  | Toe-walking | | 8 | | (61.5) | |  |
| Gastrointestinal and feeding | | | 12 | | (92.3) | |  |
|  | Chronic constipation | | 8 | | (61.5) | |  |
|  | Chronic vomiting | | 1 | | (7.7) | |  |
|  | Colon polyps | | 1 | | (7.7) | |  |
|  | Elevated liver enzymes | | 1 | | (7.7) | |  |
|  | Fatty liver disease | | 1 | | (7.7) | |  |
|  | Feeding difficulties | | 10 | | (76.9) | |  |
|  | Ankyloglossia | | 2 | | (15.4) | |  |
|  | Choking with feeds | | 2 | | (15.4) | |  |
|  | Difficulty latching | | 7 | | (53.8) | |  |
|  | Difficulty lateralizing food | | 1 | | (7.7) | |  |
|  | Dysphagia | | 3 | | (23.1) | |  |
| **Health category / condition** | | *n* | | | (%) | |  |
|  | G-tube | | 2 | | (15.4) | |  |
|  | Hyperphagia | | 1 | | (7.7) | |  |
|  | NG-tube | | 3 | | (23.1) | |  |
|  | Oral motor hypotonia | | 5 | | (38.5) | |  |
|  | Pocketing of food | | 1 | | (7.7) | |  |
|  | Sensory-related feeding difficulties | | 6 | | (46.2) | |  |
|  | Gastroesophageal reflux disease | | 4 | | (30.8) | |  |
|  | Inguinal hernia | | 1 | | (7.7) | |  |
|  | Jaundice | | 1 | | (7.7) | |  |
|  | Pancreatitis | | 1 | | (7.7) | |  |
| Genitourinary | | | 10 | | (76.9) | |  |
|  | Ambiguous genitalia | | 1 | | (7.7) | |  |
|  | Chronic kidney disease | | 2 | | (15.4) | |  |
|  | Stage 1 | | 1 | | (7.7) | |  |
|  | Stage 2 | | 1 | | (7.7) | |  |
|  | Differences in female/female-appearing genitalia | | 2 | | (15.4) | |  |
|  | Bicornate uterus | | 1 | | (7.7) | |  |
|  | Streak ovaries | | 1 | | (7.7) | |  |
|  | Differences in male/male-appearing genitalia | | 4 | | (30.8) | |  |
|  | Bilateral testicular hypoplasia | | 2 | | (15.4) | |  |
|  | Congenital penile torsion | | 1 | | (7.7) | |  |
|  | Cryptorchidism | | 2 | | (15.4) | |  |
|  | Hypospadias | | 1 | | (7.7) | |  |
|  | Micropenis | | 1 | | (7.7) | |  |
|  | Müllerian duct | | 1 | | (7.7) | |  |
|  | Nephrogenic rest(s) (NR) | | 4 | | (30.8) | |  |
|  | Proteinuria | | 1 | | (7.7) | |  |
| **Health category / condition** | | | | *n* | (%) | |  |
|  | Renal cyst(s) | | 3 | | (23.1) | |  |
|  | Renal hypoplasia | | 1 | | (7.7) | |  |
|  | Wilms tumor (WT) | | 6 | | (46.2) | |  |
|  | One lifetime WT | | 5 | | (38.5) | |  |
|  | Two lifetime WTs | | 1 | | (7.7) | |  |
|  | Stage identified | |  | |  | |  |
|  | Stage I | | 6 | | (46.2 | |  |
|  | Stage III | | 1 | | (7.7) | |  |
|  | WT or NR treatment | |  | |  | |  |
|  | Chemotherapy | | 6 | | (46.2 | |  |
|  | Radiation | | 2 | | (15.4) | |  |
|  | Surgery | | 7 | | (53.8) | |  |
| Musculoskeletal | | | 5 | | (38.5) | |  |
|  | Fracture | | 2 | | (15.4) | |  |
|  | Low vitamin D | | 1 | | (7.7) | |  |
|  | Multiple hereditary exostoses | | 1 | | (7.7) | |  |
|  | Osteopenia | | 1 | | (7.7) | |  |
|  | Scoliosis | | 1 | | (7.7) | |  |
|  | Shortened calf muscles | | 1 | | (7.7) | |  |
| Neurological | | | 13 | | (100.0) | |  |
|  | Abnormal brain MRI | | 3 | | (23.1) | |  |
|  | Epilepsy | | 1 | | (7.7) | |  |
|  | Hypotonia | | 7 | | (53.8) | |  |
|  | Hypotonia and hypertonia (mixed) | | 3 | | (23.1) | |  |
|  | Insensitivity to pain / high pain tolerance | | 10 | | (76.9) | |  |
|  | Macrocephaly | | 1 | | (7.7) | |  |
|  | Microcephaly | | 3 | | (23.1) | |  |
| **Health category / condition** | | | | *n* | (%) | |  |
|  | Plagiocephaly | | 3 | | (23.1) | |  |
|  | Spastic diplegic cerebral palsy | | 1 | | (7.7) | |  |
|  | Spasticity | | 1 | | (7.7) | |  |
| Ocular | | | 13 | | (100.0) | |  |
|  | Amblyopia | | 1 | | (7.7) | |  |
|  | Aniridia | | 13 | | (100.0) | |  |
|  | Aphakia | | 4 | | (30.8) | |  |
|  | Astigmatism | | 1 | | (7.7) | |  |
|  | Cataract(s) | | 11 | | (84.6) | |  |
|  | Corneal clouding | | 1 | | (7.7) | |  |
|  | Corneal keratopathy/pannus | | 2 | | (15.4) | |  |
|  | Foveal/macular hypoplasia | | 2 | | (15.4) | |  |
|  | Glaucoma | | 10 | | (76.9) | |  |
|  | Myopia | | 3 | | (23.1) | |  |
|  | Nystagmus | | 12 | | (92.3) | |  |
|  | Optic nerve hypoplasia | | 3 | | (23.1) | |  |
|  | Peter's anomaly | | 1 | | (7.7) | |  |
|  | Ptosis | | 1 | | (7.7) | |  |
|  | Retinal detachment | | 2 | | (15.4) | |  |
|  | Strabismus | | 3 | | (23.1) | |  |
| Respiratory | | | 5 | | (38.5) | |  |
|  | Asthma | | 3 | | (23.1) | |  |
|  | Pneumonia | | 4 | | (30.8) | |  |
|  | Recurrent pneumonia | | 2 | | (15.4) | |  |
|  | Respiratory tract infections requiring antibiotics | | 3 | | (23.1) | |  |
